# An amalgamated risk estimation model (REM) and assay integration into future REMs

**DOI:** 10.1186/gb-2011-12-s1-p37

**Published:** 2011-09-19

**Authors:** Peter Cartwright, Erica Ramos, India Bradley, Eric Hanson

**Affiliations:** 1 Advanced Medical Imaging and Genetics (Amigenics), 5495 South Rainbow Boulevard, Suite 102, Las Vegas, NV 89118, USA

## 

The clinical reality of the post-genomic era is that we now face even more complex disease processes when provided with genomic information, including multifactorial genetic and genomic influences, and epigenetic and environmental factors. A useful example of the promise and perils of genomic technologies and information is breast cancer. By the mid-1990s, two genes (*BRCA1* and *BRCA2*) had been identified, accounting for approximately 5% of affected individuals. Since then, surprisingly few genetic breast cancer risk factors have been identified to account for the remaining 95%. To efficiently and cost-effectively identify individuals at high risk, a combination of information components is required: a patient-reported personal and family medical history; clinical data (for example, a physical exam, pathology results, laboratory test results and imaging); and genetic/genomic results. Gaining comprehensive data from all of these areas provides the best risk assessment and management options for patients. Furthermore, high quality patient and clinical information is essential for the accurate and reliable interpretation of genomic results.

We have clinically implemented a platform that integrates all three informational components with multiple risk estimation models (REMs) to produce an effective automated method for risk-stratifying patients. Although this platform can be and has been applied to a wide range of genetic conditions, this presentation will use breast cancer to illustrate the approach. This system consists of three primary components: a secure web-based questionnaire used by patients to enter personal and family medical history; a tablet-based system for collecting clinical and genomic information; and an analysis engine that seamlessly integrates REMs that have been developed to calculate either a woman’s risk of developing breast cancer during her lifetime (Claus, Gail II, BRCAPRO, BOADICEA and IBIS) or the probability of detecting a hereditary breast cancer gene mutation (Myriad, Penn II, BRCAPRO, BOADICEA and IBIS).This use of multiple or amalgamated REM (aREM) results offers one of the most comprehensive breast cancer risk assessments available for predicting the lifetime risk of developing breast cancer or the presence of *BRCA* mutations. Additional uses for aREMs include rapid analyses of existing breast cancer datasets, external validation of new REMs, and prospective outcome comparisons based on initial aREM results.

Numerous biomarkers for breast cancer, in addition to *BRCA1* and *BRCA2* mutations, have been reported, but few molecular markers or assays have been adopted for clinical use. The addition of novel REMs that integrate a new molecular assay or classifiers can facilitate the identification of an enriched population for screening (for example, lowering the number needed to screen) or for diagnostic, prognostic or therapeutic purposes. REMs are rapidly integrating multiple genetic influences, whole genome sequencing data and epigenetic modifications, so structured comparisons of the performance of existing and emerging predictive REMs are required for safe and effective clinical application.

